# Correction: Anti-ulcerogenic effect of KFP-H008 against ethanol-induced gastric ulcer *via* p38 MAPK/NF-κB pathway

**DOI:** 10.1039/c9ra90042j

**Published:** 2019-06-04

**Authors:** Mei Su, Cheng-yuan Li, Lin Zhou, Yun-yi Yan, Lu-yao Ao, Guang-ji Wang, Wei-rong Fang, Yun-man Li

**Affiliations:** State Key Laboratory of Natural Medicines, School of Pharmacy, China Pharmaceutical University Nanjing 210009 PR China guangjiwang@hotmail.com; State Key Laboratory of Natural Medicines, School of Basic Medicine and Clinical Pharmacy, China Pharmaceutical University Nanjing 210009 PR China weirongfang@163.com yunmanlicpu@hotmail.com +86 2583271173 +86 2583271173

## Abstract

Correction for ‘Anti-ulcerogenic effect of KFP-H008 against ethanol-induced gastric ulcer *via* p38 MAPK/NF-κB pathway’ by Mei Su *et al.*, *RSC Adv.*, 2017, **7**, 49423–49435.

The authors regret that the email address was not included for one of the three corresponding authors, Guang-ji Wang. Contact email addresses for all three corresponding authors are presented here.

The Royal Society of Chemistry apologises for these errors and any consequent inconvenience to authors and readers.

## Supplementary Material

